# Supplementary material for: Tissue-Specificity of Gene Expression Diverges Slowly between Orthologs, and Rapidly between Paralogs
Source: PLoS Comput Biol. 2016 Dec 28;12(12):e1005274. doi: 10.1371/journal.pcbi.1005274 (PMC5193323; doi:10.1371/journal.pcbi.1005274)
Supplement: S1 Fig — (DOCX) [file pcbi.1005274.s001.docx]

**Supplemental Figures**

**Legend for figures:**

Fig A – Fig N and Fig T: X-axis, divergence time in million years between the genes compared; Y-axis, Pearson correlation between values of τ over genes. In red, the correlation of orthologs between the focal species and other species; representative species are noted above the figure; there are several points when there are several datasets for a same species; the size of red circles is proportional to the number of tissues used for calculation of tissue specificity. In blue, the correlation of paralogs in the focal species, according to the date of duplication; representative taxonomic groups for this dating are noted under the figure; the size of blue circles is proportional to the number of genes in the paralog group.

Fig Q – Fig S: Each bar represents the number of gene pairs of a given type for a given phylogenetic age, for which both genes of the pair are tissue-specific. In dark colour, the number of gene pairs specific of the same tissue; in light colour, the number of gene pairs specific of different tissues. Orthologs are in red, in the left panel, paralogs are in blue, on the right panel; notice that the scales are different for orthologs and for paralogs. The overall proportions of pairs in the same or different tissues are indicated for orthologs and paralogs; in addition, for paralogs the proportion for pairs younger than the divergence of tetrapods is also indicated.

**Fig A: Pearson correlation of tissue specificity according to human Bodymap dataset.**

**Fig B: Pearson correlation of tissue specificity according to human Fageberg dataset.** Only conserved orthologs (up to frog, present in all analysed species).

**Fig C: Pearson correlation of tissue specificity according to human Bodymap dataset.** Only conserved orthologs (up to frog, present in all analysed species).

**Fig D: Pearson correlation of tissue specificity according to mouse dataset.** Only conserved orthologs (up to frog, present in all analysed species).

**Fig E: Pearson correlation of tissue specificity according to human Fagerberg dataset.** Tissue-specificity calculated without testis.

**Fig F: Pearson correlation of tissue specificity according to human Bodymap dataset.** Tissue-specificity calculated without testis.

**Fig G: Pearson correlation of tissue specificity according to mouse dataset.** Tissue-specificity calculated without testis.

**Fig H: Pearson correlation of tissue specificity according to mouse dataset.** Tissue-specificity calculated without brain.

**Fig I: Pearson correlation of tissue specificity according to mouse dataset.** Tissue-specificity calculated without heart.

**Fig J: Pearson correlation of tissue specificity according to mouse dataset.** Tissue-specificity calculated without kidney.

**Fig K: Pearson correlation of tissue specificity according to mouse dataset.** Tissue-specificity calculated without liver.

**Fig L: Pearson correlation of tissue specificity according to human Fagerberg dataset.** Tissue-specificity calculated without sex-chromosome genes.

**Fig M: Pearson correlation of tissue specificity according to human Bodymap dataset.** Tissue-specificity calculated without sex-chromosome genes.

**Fig N: Pearson correlation of tissue specificity according to mouse dataset.** Tissue-specificity calculated without sex-chromosome genes.

**Fig O: Distribution of tissue-specificity between orthologs and paralogs.**

**Fig P: Distribution of tissue-specificity in paralogs of different age of duplication.**

**Fig Q: Difference of tissue-specificity between orthologs and paralogs.** Tau cut-off 0.8 and calculated without testis.

**Fig R: Difference of tissue-specificity between orthologs and paralogs.** Tau cut-off 0.3.

**Fig S: Difference of tissue-specificity between orthologs and paralogs.** Tau cut-off 0.3 and calculated without testis.

**Fig T: Pearson correlation of tissue specificity according to human Fagerberg dataset.** Tissue-specificity calculated without tissue-specific genes (Tau > 0.8).


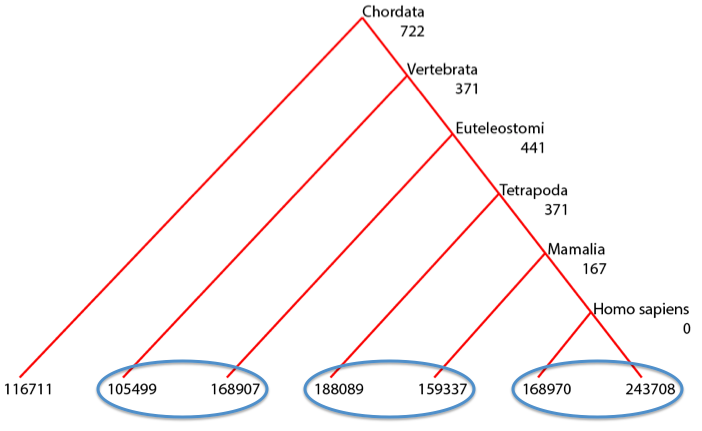


**Fig U: Choice of paralogs.** The tree is the example for one paralog family. The blue circles represent how the youngest couple of paralogs was chosen for different phylogenetic ages. Gene names are of the form ENSG00000xxxxxx, with xxxxxx to be replaced with the numbers shown on the figure.


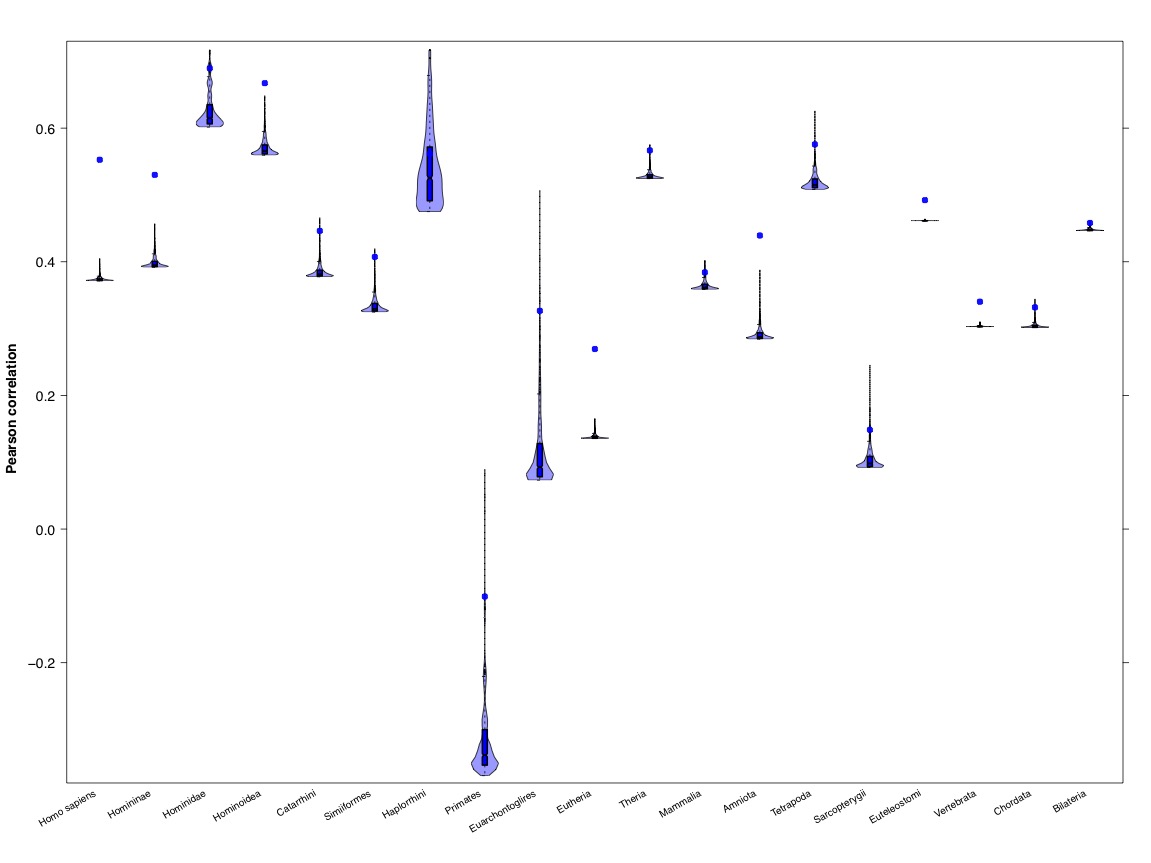


**Fig V: Pearson correlations between paralogs.** Box plots represents 1000 random attribution of paralogs in each pair to the x and y vectors for the correlation. The blue dot is the correlation between the paralogs sorted as in the main analysis, i.e. the highest expressed in x and the lowest in y for each pair.
