# Supplementary material for: Tissue-Specificity of Gene Expression Diverges Slowly between Orthologs, and Rapidly between Paralogs
Source: PLoS Comput Biol. 2016 Dec 28;12(12):e1005274. doi: 10.1371/journal.pcbi.1005274 (PMC5193323; doi:10.1371/journal.pcbi.1005274)
Supplement: S1 Table — (DOCX) [file pcbi.1005274.s002.docx]

**Supplementary Tables**

**Table A:** Number of protein coding genes used for the analysis.

| **Organisms/data sets** | Fagerberg | Brawand | Bodymap | ENCODE | Necsulea | Merkin | Keane |
| --- | --- | --- | --- | --- | --- | --- | --- |
| Human | 18569 | 19151 | 19113 |  |  |  |  |
| Gorilla |  | 17069 |  |  |  |  |  |
| Chimp |  | 16507 |  |  |  |  |  |
| Macaca |  | 18297 |  |  |  | 19749 |  |
| Mouse |  | 18086 |  | 19442 |  | 18538 | 16892 |
| Rat |  |  |  |  |  | 19215 |  |
| Cow |  |  |  |  |  | 17634 |  |
| Opossum |  | 16622 |  |  |  |  |  |
| Platypus |  | 19036 |  |  |  |  |  |
| Chicken |  | 14332 |  |  |  | 14780 |  |
| Frog |  |  |  |  | 15499 |  |  |
| Fly |  |  |  | 10960 |  |  |  |

**Table B:** Number of one-to-one orthologous genes of organisms to human, used for the main analysis.

| **Organisms/data sets** | Fagerberg | Brawand | Bodymap | ENCODE | Necsulea | Merkin | Keane |
| --- | --- | --- | --- | --- | --- | --- | --- |
| Human | - | 17170 | 17224 |  |  |  |  |
| Gorilla |  | 14813 |  |  |  |  |  |
| Chimp |  | 15282 |  |  |  |  |  |
| Macaca |  | 14578 |  |  |  | 14943 |  |
| Mouse |  | 14397 |  | 14876 |  | 14791 | 14056 |
| Rat |  |  |  |  |  | 14040 |  |
| Cow |  |  |  |  |  | 14666 |  |
| Opossum |  | 12445 |  |  |  |  |  |
| Platypus |  | 10490 |  |  |  |  |  |
| Chicken |  | 11352 |  |  |  | 11525 |  |
| Frog |  |  |  |  | 11462 |  |  |
| Fly |  |  |  | 2750 |  |  |  |
